# Giant Brunner’s gland hamartoma: the surgical management of complex cases

**DOI:** 10.1308/rcsann.2025.0122

**Published:** 2026-01-20

**Authors:** P Sen, T Broquetas, M Pera

**Affiliations:** ^1^ King’s College London, UK; ^2^ Hospital del Mar, Barcelona, Spain

**Keywords:** Brunner’s gland, Capsule endoscopy, Minimally invasive surgery, Conversion to an open surgical procedure

## Abstract

Brunner’s gland hamartoma (BGH) is a rare, benign tumour composed of mucin-secreting glands within the duodenal submucosa. Lesions typically range from 0.5 to 1.0cm and are usually managed endoscopically, with larger tumours occurring infrequently. This case involves a 74-year-old woman presenting with syncope. Diagnostic investigations revealed a massive BGH with a 10cm pedicle, accompanied by significant iron deficiency anaemia and intussusception. Although there are no formal, universally established guidelines, literature favours endoscopic removal because of reduced bleeding, a shorter hospital stay and lower cost. However, open surgical resection proved more appropriate owing to the lesion’s size and complexity. This case highlights the challenges of managing giant BGHs and supports open surgical intervention when standard methods are unsuccessful.

## Background

Brunner’s glands lie primarily in the proximal duodenum, decreasing distally along the submucosa. These glands are responsible for secreting alkaline mucus that protects the duodenal lining from the acidic contents of the stomach, thus playing a vital role in maintaining mucosal integrity and facilitating digestion. Mainly affecting patients in their fifth and sixth decade of life, Brunner’s gland hamartoma (BGH) represents fewer than 1.0% of all primary tumour cases in the small bowel, with rare malignant progression to adenocarcinoma.^1,2^ The scarcity of these lesions and their mostly benign nature contribute to the limited number of cases reported in the literature.

This case involves a giant hamartoma with a diameter greater than 4cm and a large pedicle of 10cm. Most lesions range in diameter between 0.5 and 1.0cm and very rarely are larger than 5cm, making this case a particularly intriguing manifestation.^3^ The sheer size and morphology of such lesions can pose significant diagnostic and therapeutic challenges. Many cases are asymptomatic and diagnosed incidentally when performing imaging or endoscopic studies, underscoring the often silent nature of this condition. In rare cases, complications such as large haemorrhages, intestinal obstructions or intussusception can be observed, as seen in this case.^4^ These complications arise primarily because of the size, location and pedunculated nature of the lesion, which can cause mechanical obstruction or mucosal ulceration leading to bleeding.

There are no formal, universally established guidelines from major gastroenterological or surgical societies owing to its rarity. This absence of consensus guidelines presents challenges in standardising care. However, management principles have emerged in recent years from case reports, highlighting the need for further case reporting to increase visibility, and subsequent clinical studies. This is essential to improving understanding, refining diagnostic criteria, and optimising therapeutic approaches for BGH.

## Case report

A 74-year-old woman with a past medical history of hypertension and type II diabetes mellitus, and surgical history of sigmoidectomy in 2008 for a colonic adenocarcinoma, was referred to the gastroenterologist by the patient’s general practitioner to investigate iron deficiency anaemia detected in January 2017, following an episode of syncope. Her haemoglobin (Hb) level was 9.5g/dL with low iron and ferritin levels. She reported having undergone an upper gastrointestinal endoscopy and fibreoptic colonoscopy elsewhere with normal results.

Despite treatment with oral iron, her Hb level persisted at 10g/dL, indicating ongoing blood loss or malabsorption. A capsule endoscopy and an abdominal computed tomography (CT) scan were both recommended as diagnostic studies, but capsule endoscopy was performed first for scheduling issues. As shown in Figure 1, a giant polyp in the small bowel was visualised on capsule endoscopy examination. The CT scan revealed a duodenal polyp about 4cm in diameter originating in the first duodenal portion, with a long pedicle of approximately 10cm, the distal ending of which reached the third duodenal portion (Figure 2). An endoscopic polypectomy was essayed by double-balloon enteroscopy, albeit unsuccessful because of the polyp size, indicating the need for surgical intervention. This highlights the limitations of minimally invasive approaches in cases involving large or technically challenging lesions.

**Figure 1:**
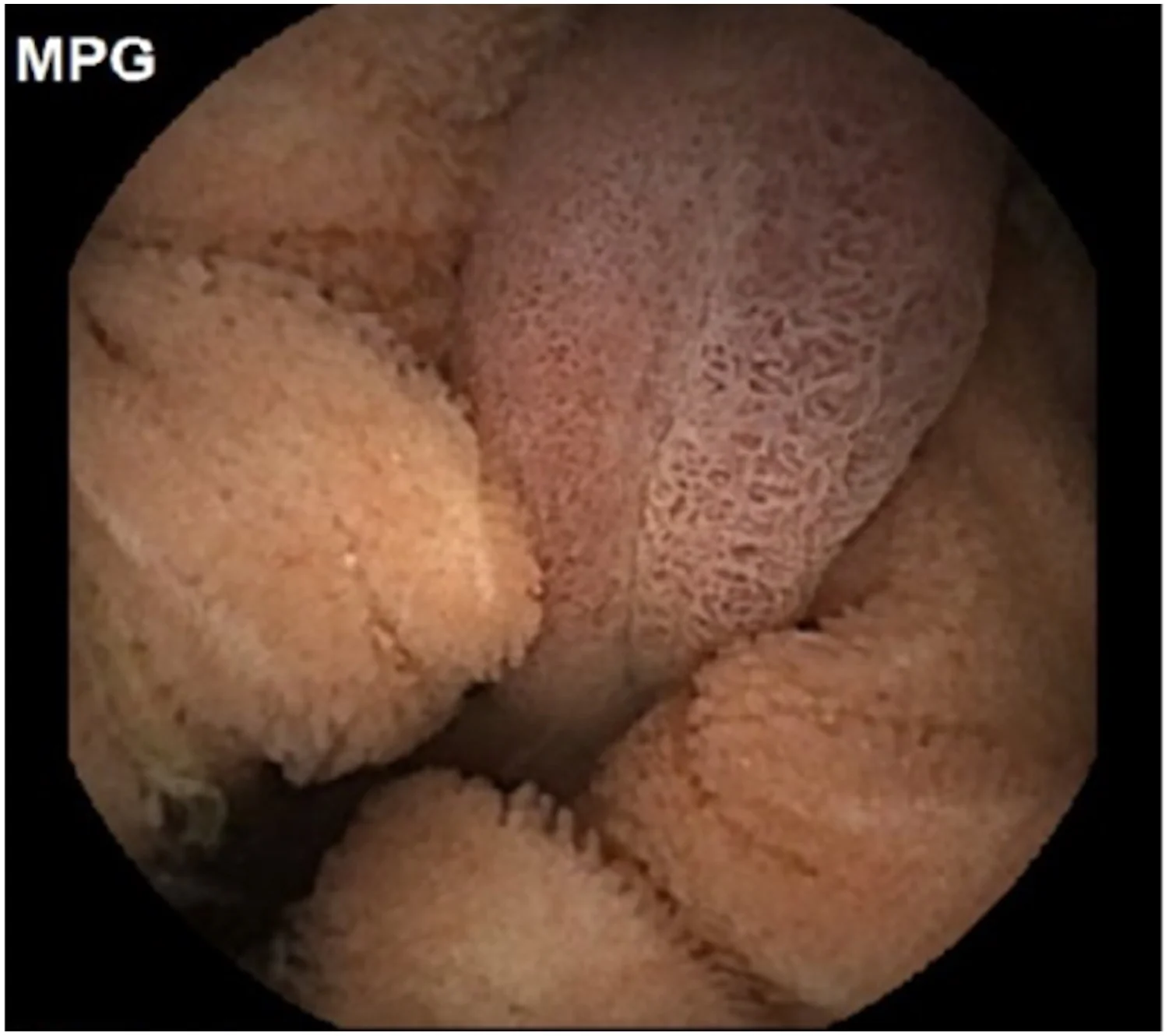
Capsule endoscopy image of the Brunner’s gland hamartoma pedicle

**Figure 2:**
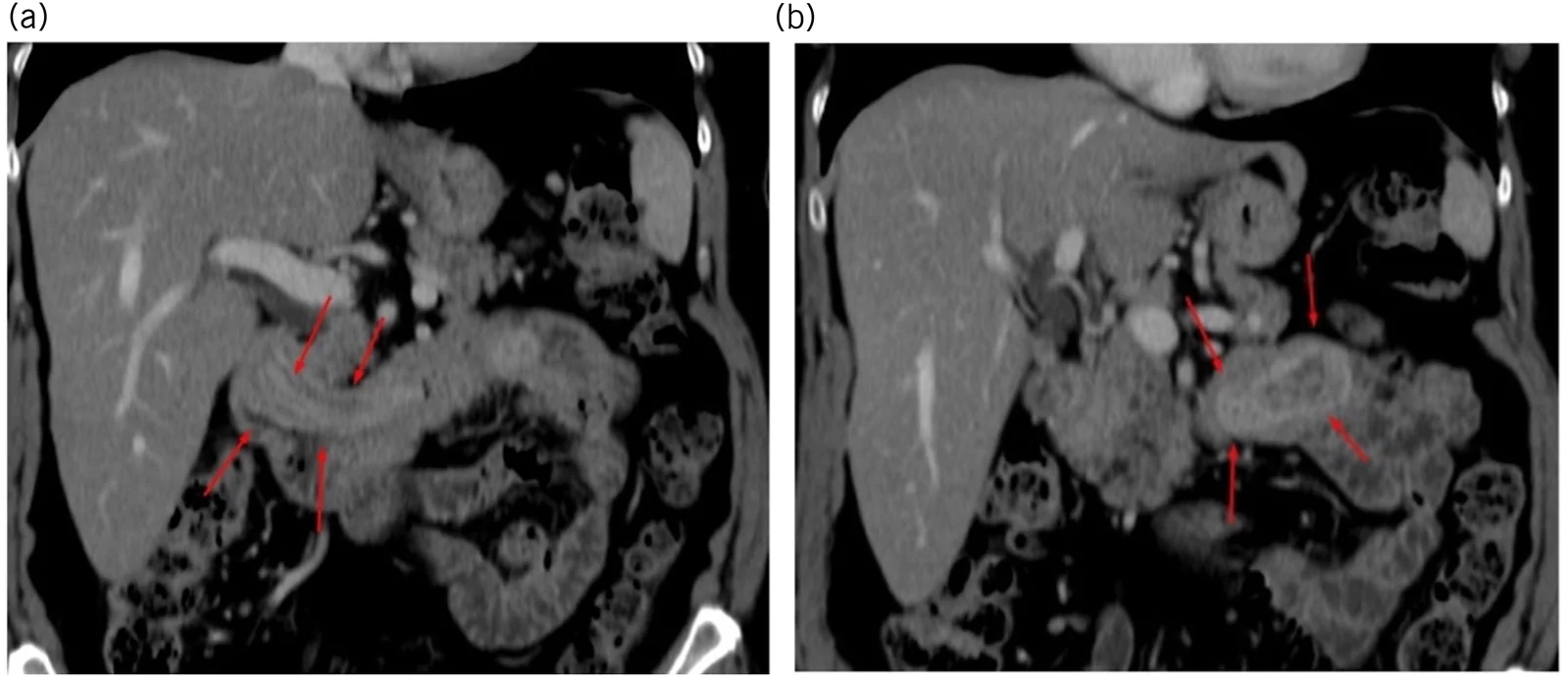
Computed tomography (CT) imaging of Brunner’s gland hamartoma. (a) CT scan showing the 10cm base of the pedunculated hamartoma originating from the first portion of the duodenum (red arrows indicate the polyp base). (b) CT scan highlighting the head of the polyp, greater than 4cm in diameter within the duodenum (red arrows indicate the polyp head).

Using an open surgical approach, the lesion was identified with an area of intussusception and its origin was located 2cm distal to the pylorus. An enterotomy was performed through the pylorus and the duodenum, clearly evidencing the large polyp (Figure 3). We proceeded to resection with posterior pyloroplasty and pathological examination revealed Brunner’s glands with no signs of malignancy. The surgical approach allowed direct access and control, especially important in complicated cases involving intussusception and haemorrhage.

**Figure 3:**
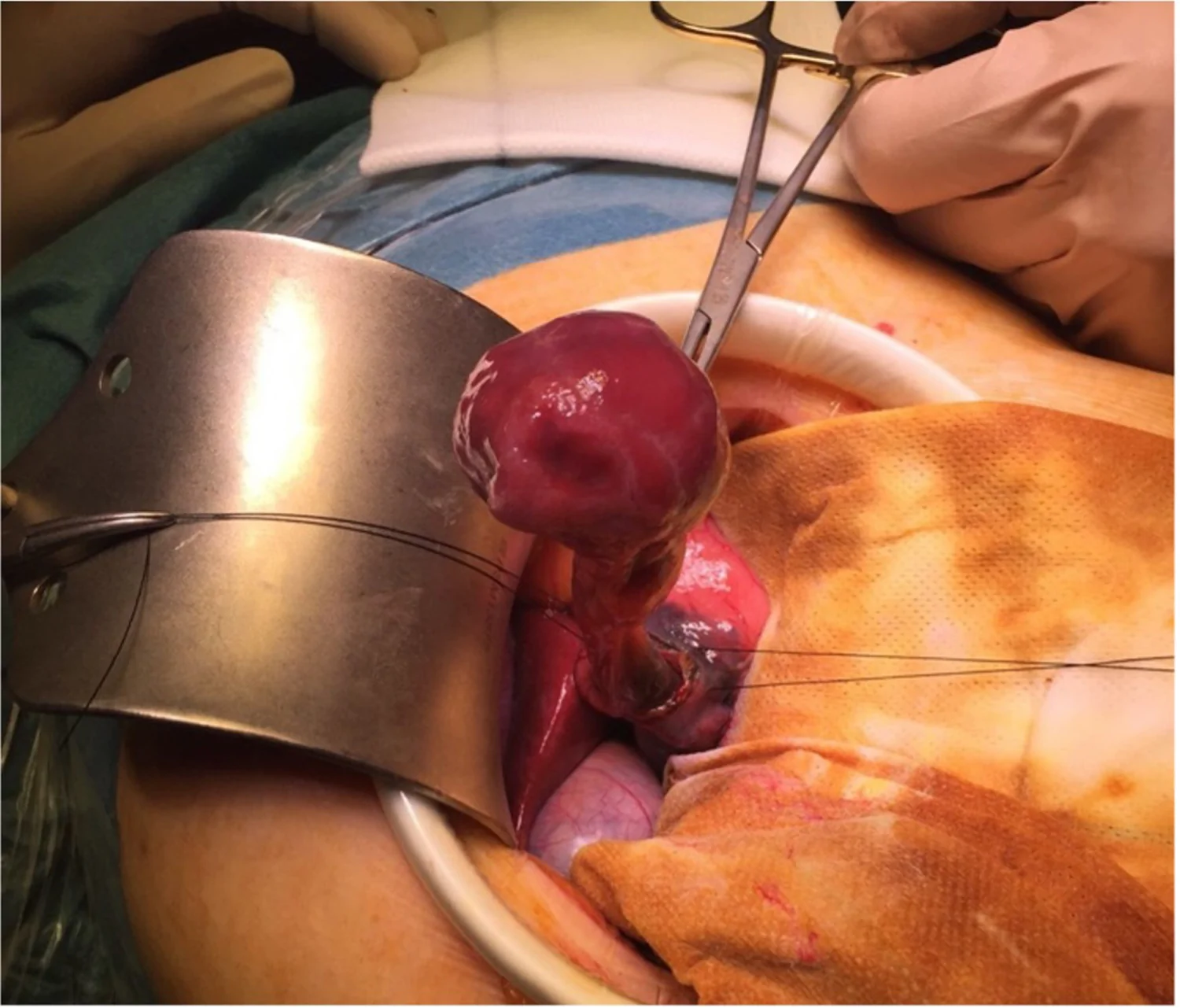
Intraoperative image of the Brunner’s gland hamartoma in the first portion of the duodenum

Postoperatively, examination demonstrated a soft, non-tender abdomen and excellent pain control with epidural analgesia. The patient developed a seroma during the immediate postoperative period that healed rapidly, leading to discharge within 2 weeks. The follow-up appointment 6 months after the duodenotomy revealed normal abdominal examination and full recovery. This outcome underlines the effectiveness and safety of the chosen surgical approach in this clinical scenario.

## Discussion

BGH can be differentiated from hyperplasia by the size of the lesion. Masses less than 0.5cm are identified as Brunner’s hyperplasia and are often asymptomatic.^1^ Although the histological differences between hamartomatous and hyperplastic lesions are not well understood, it is thought that the combination of proliferated smooth muscle with cystically dilated glands in the submucosa differentiates BGH from hyperplasia.^5^ This distinction is important, because it guides clinical management and prognosis.

As with much of the research surrounding BGH, the aetiology is still unclear. However, several studies associate BGH with an increase in gastric secretion, potentially by *Helicobacter pylori* infection, including the study by I. Kovacević *et al*, which found that 71% of patients with BGH had concurrent *H. pylori* infections.^6,7^ It is hypothesised that the increased acidic gastric environment stimulates mucin production, which in turn alkalises the duodenal fluid and promotes hyperplasia of the Brunner’s glands.^8^ This proposed mechanism offers a plausible explanation linking gastric acidity, bacterial infection and glandular proliferation, although further research is warranted to clarify causation.

Capsule endoscopy is widely used to evaluate small bowel bleeding or obscure lesions, but no specific reports exist documenting its use in diagnosing BGH in the duodenum. This non-invasive modality can be used to visualise the entire small intestine and to identify lesions beyond the reach of standard endoscopy. In the current case, both capsule endoscopy and CT were useful diagnostic studies, but the size and shape of the lesion were clearly seen on radio-imaging examination. BGH discovered on CT have been rarely reported, with only 7 cases in a review of 26 patients from the radiologic pathology archive at the Armed Forces Institute of Pathology.^9^ Although there are no formal guidelines for the management of BGH, much of the current literature suggests using endoscopic resection as first-line treatment.^10^ In this case, both attempts at double-channel gastroscopy and duodenoscopy were insufficient, requiring conversion to open surgery. Owing to the lesion’s considerable size, absence of universal management guidelines, associated intussusception and haemorrhage, as well as the challenges in submucosal penetration on endoscopy and unsuccessful minimally invasive attempts, the multidisciplinary team was required to demonstrate resilience, clinical adaptability and innovative decision making. This case exemplifies the importance of flexibility and teamwork in managing rare and complex cases.

## Conclusion

Management of a massive BGH presenting with anaemia, haemorrhage and intussusception requires thorough investigation and careful evaluation to ensure optimal patient outcomes and recovery. We would like to reinforce this method of duodenotomy, polyp resection and pyloroplasty in the surgical management of large BGH and we hope that our findings can aid and shape the management of BGH in atypical cases. Ongoing documentation and analysis of similar cases will be critical in forming more concrete guidelines for future clinicians facing this rare but challenging entity.

## Conflicts of interest

The author/s declare no competing interests.

## Funding

The author/s received no financial support for the research, authorship and/or publication of this article.

## Ethics approval

Not applicable.

## Consent to participate statement

Written patient consent has been gained according to the Royal College of Surgeons Annals Guidelines.

## Artificial Intelligence

The author/s declare that no AI was used to conduct the study or prepare the manuscript.
